# Shape of growth plate of proximal femur in children and its significance in the aetiology of slipped capital femoral epiphysis

**DOI:** 10.1007/s00264-012-1699-y

**Published:** 2012-11-09

**Authors:** Grzegorz Kandzierski, Łukasz Matuszewski, Anna Wójcik

**Affiliations:** Children’s Orthopaedic Clinic and Rehabilitation Department, Medical University of Lublin, Lublin, Poland

## Abstract

**Purpose:**

The main objective of the study was to present the influence of the morphological shape of the proximal femoral growth plate in children as one of the risk factors for the incidence of slipped capital femoral epiphysis (SCFE) in adolescents.

**Methods:**

This research is based on the X-ray, computed tomography (CT) and magnetic resonance imaging (MRI) data obtained for 100 children three to 13 years old, all treated at the Children’s Orthopaedic Clinic and Rehabilitation Department and Department of Radiology Medical University of Lublin between 2005 and 2009. We took into account 83 children with healthy hip joints and 17 children with SCFE. We also performed morphological analysis of the shape of the proximal femoral growth plate together with X-ray, CT and MRI examinations of the proximal ends of cadaver femurs for two children aged six and 13.

**Results:**

In the final findings we present an analysis of the shape of the proximal femoral growth plate in children between the third and 13th years of life and consider a correlation between the shape of the proximal femoral growth plate and its influence on the incidence of SCFE in adolescents.

**Conclusions:**

The change of shape of the proximal femoral growth plate from pleated to more spherical is one of the risk factors for the incidence of SCFE in children ten years old and older.

## Introduction

Slipped capital femoral epiphysis (SCFE) is a very specific type of disease where the head of the femur slips off the neck of the thigh bone. The cause of SCFE is still unknown. Risk factors that increase the likelihood of SCFE include obesity, pubertal growth spurt, medications, thyroid disease, radiation or chemotherapy treatment and bone problems related to kidney disease [1–5]. The ultimate goal in SCFE treatment is to diagnose the condition as early as possible in order to prevent the head of the femur from slipping further, thus preventing hip deformity [6, 7]. Severe complications are usually caused by a delayed diagnosis. Research at various medical centres throughout the world continue to investigate SCFE in efforts to improve treatment and prevention of this condition [8–13]. We want to emphasise the mechanical hypotheses, which have been proposed by our clinic to explain this phenomenon. Because the growth plate appears to be most vulnerable to shear stress and injury in children, SCFE never occurs in a mature person once the growth plate has closed.

The growth plate is sandwiched between the epiphysis and the metaphysis and connects the neck of a femur to the head. The growth plate is made of a special type of cartilage that builds bone on the top end of the metaphysis. The periphery of the physis consists of two elements: the groove of Ranvier and the perichondrial ring of LaCroix [14, 15]. The perichondrial ring is a dense fibrous structure that surrounds the physis and provides stability to the growth plate. It consists of vertical, horizontal and oblique collagen fibres which are very resistant to shearing forces. Wong-Chung et al. [8] obtained hips post-mortem from children five days to 15 years old and tested them to failure for shear strength. They noticed the importance of the fibrocartilaginous ring as a supporting structure.

Serafini-Fracassini and Smith [16] focused on the analysis of the acting shear and the direction of stress forces, and obliquity of the proximal femoral growth plate. They suggested that shear and stress forces are always perpendicular to obliquity of the proximal femoral growth plate. The explanation of the phenomena that can influence the shape and architecture of the growth plate has been provided by “Wolff’s law”. Moreover, Pazzaglia et al. [17] noticed that in growing animals this particular theory is supported not only by developmental studies but also by clinical experience.

SCFE can develop for numerous reasons and the shape of the growth plate might be an important factor that increases chances for its occurrence. However, it is still a conundrum why SCFE almost never occurs in children under ten years of age.

### Purpose of the study

The main objective of the study was to present the influence of the morphological shape of the proximal femoral growth plate in children as one of the risk factors for SCFE in adolescents. In addition, we want to show the changes of dimensional shaping of the proximal femoral growth plate over time.

## Methods

This research is based on the X-ray, computed tomography (CT) and magnetic resonance imaging (MRI) data obtained for 100 children three to 13 years old, all treated at the Children’s Orthopaedic Clinic and Rehabilitation Department and Department of Radiology Medical University of Lublin between 2005 and 2009. We took into account 83 children with healthy hip joints and 17 children with SCFE (Tables 1 and 2). We also performed a morphological analysis of the shape of the proximal femoral growth plate together with X-ray, CT and MRI examinations of the proximal ends of cadaver femurs for two children aged six and 13.

**Table 1:** Patients’ age, type and number of exams of healthy hip joints

Age (years)	X-ray	CT	MRI
3–4	15	0	2
5–7	20	13	18
8–10	25	14	20
11–13	23	10	9
Total	83	37	69

**Table 2:** Patients’ age, type and number of exams of hip joints with SCFE

Age (years)	X-ray	CT	MRI
3–10	0	0	0
11–13	17	0	5
Total	17	0	5

## Results

**Fig. 1:**
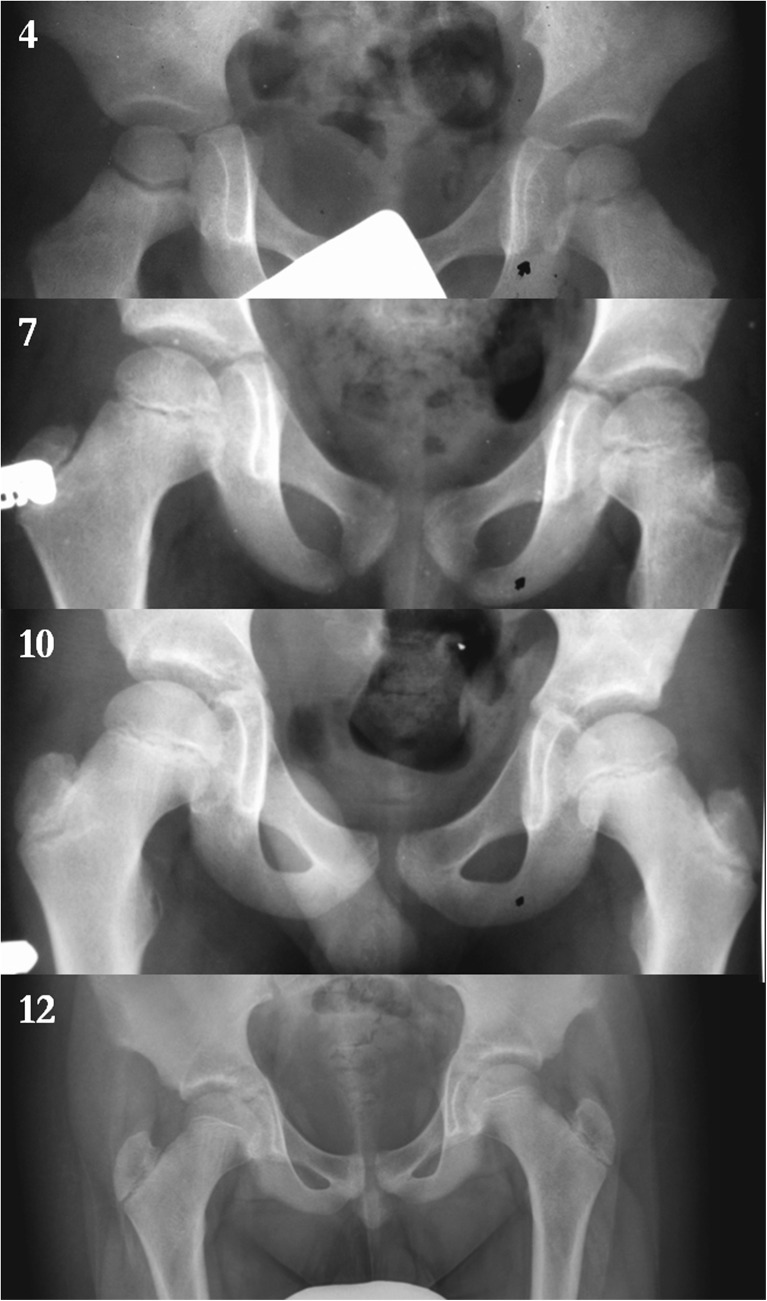
X-rays showing evolution of the growth plate of the proximal femoral epiphysis in a 4-, 7-, 10- and 12-year-old child

X-ray images demonstrate the characteristic shape of a growth plate of the proximal femoral epiphysis. In the first group we analysed 15 X-ray images for children between three and four years old. The growth plate of a proximal femoral epiphysis is demonstrated as a thick, arc-shaped and uniform radiological negative line that closely resembles a concave meniscus with epiphysis lying in it. In the second group we analysed 20 X-ray images for children between five and seven years old. In children five to six years old the growth plate of the proximal femoral epiphysis becomes flat and is depicted as a non-uniform, corrugated main line with a few tiny parallel lines. In seven year-old children the same feature looks like a straight line with a greater number of small radiological negative parallel lines. In the third group we analysed 25 X-ray images for children between eight and ten years old. In an eight year-old child the growth plate is a straight, ridged non-uniform line. In children nine to ten years old this irregular line starts to change into an arch. In the fourth group of 23 children 11–13 years old, the growth plate transforms into a thin, arc-shaped convex line. The proximal surface of it is more planar and regular (Fig. 1)

**Fig. 2:**
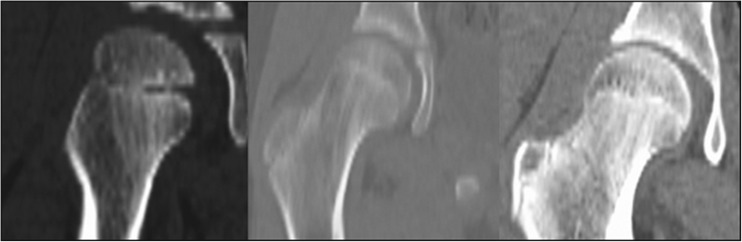
CT scan of a growth plate of the proximal femoral epiphysis in a 7-, 10- and 13-year-old child

CT images also demonstrate the shape of the growth plate of the proximal femoral epiphysis and offer a better structural definition. In the first group we analysed 13 children aged between five and seven. CT gives more details of the growth plate shape, though it still looks like a thin, irregular line. Both ends of that line start rising and the middle part is slightly lower. In the next group of 14 children between eight and ten years of age the growth plate changes its shape into an arch. In the last group we have ten children aged 11 to 13. The growth plate looks like a convex meniscus with a planar and regular proximal surface. The corrugations are gone (Fig. 2).

**Fig. 3:**
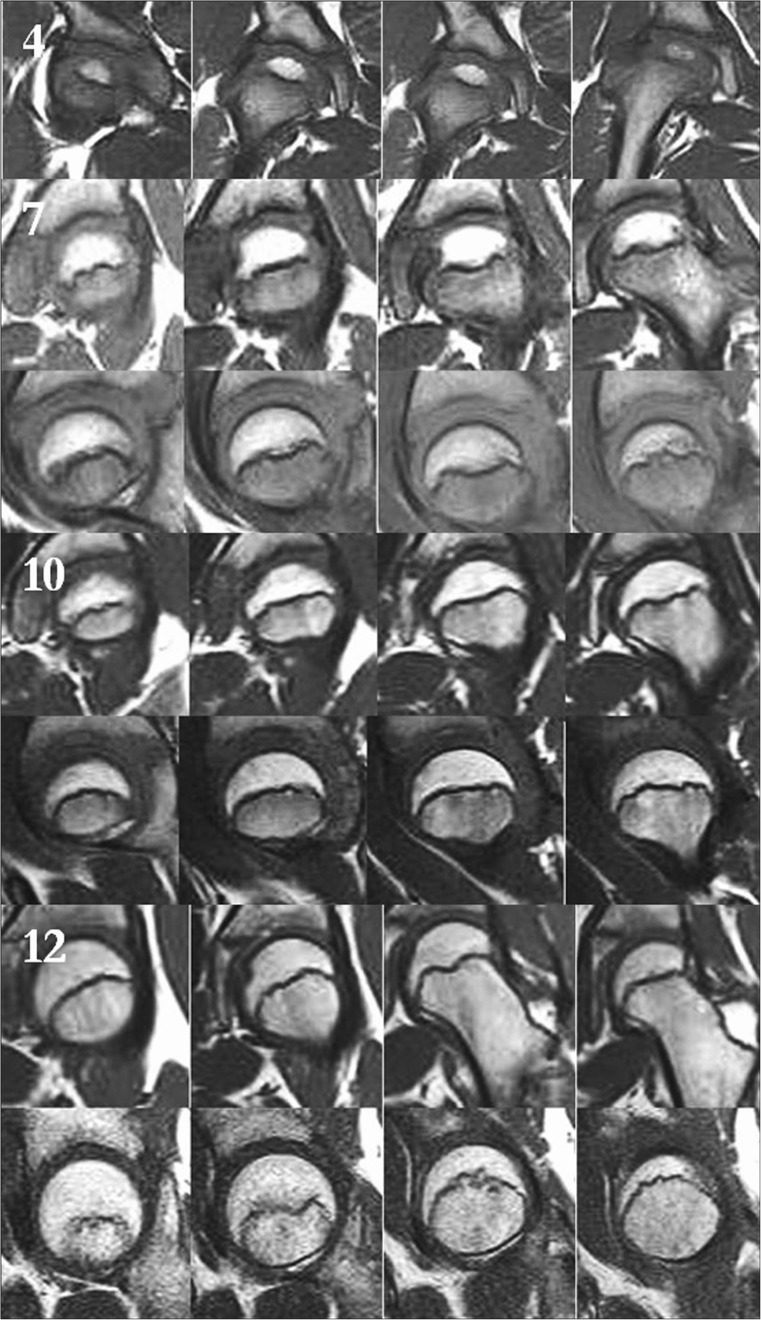
MRI scans of a growth plate of the proximal femoral epiphysis in a 4-, 7-, 10- and 12-year-old child

**Fig. 4:**
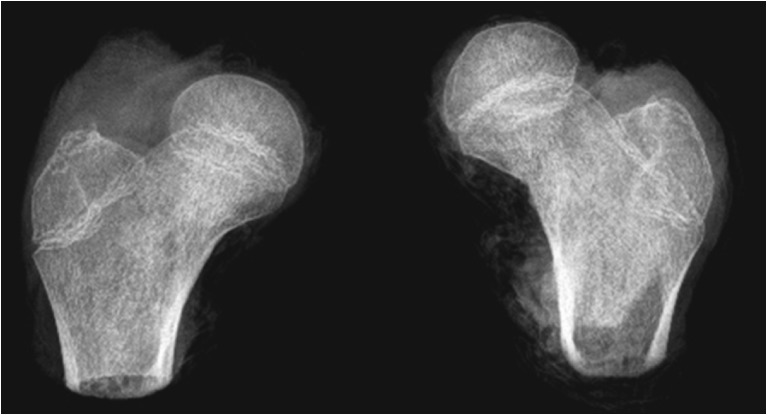
X-ray image of a growth plate of the proximal femoral epiphysis of a 6-year-old cadaver

**Fig. 5:**
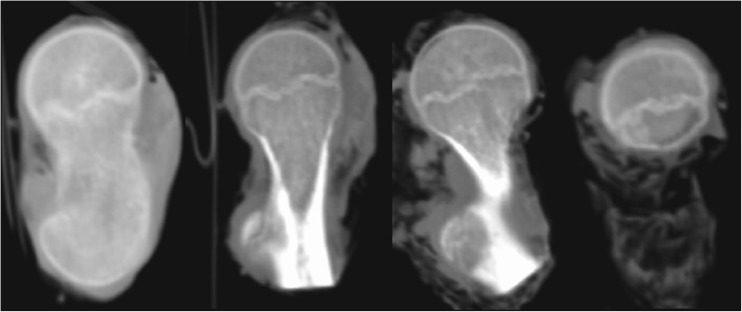
CT scans of a growth plate of the proximal femoral epiphysis of a 6-year-old cadaver

**Fig. 6:**
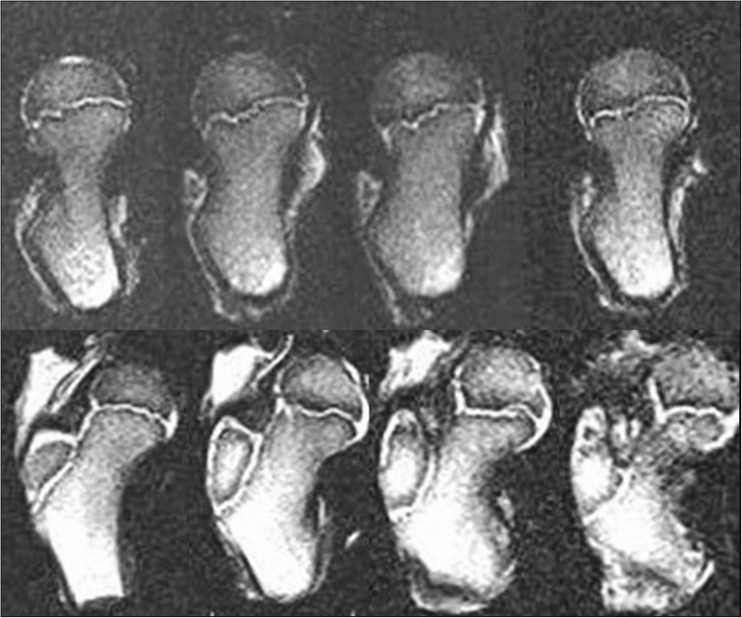
MRI scans of a growth plate of the proximal femoral epiphysis of a 6-year-old cadaver

**Fig. 7:**
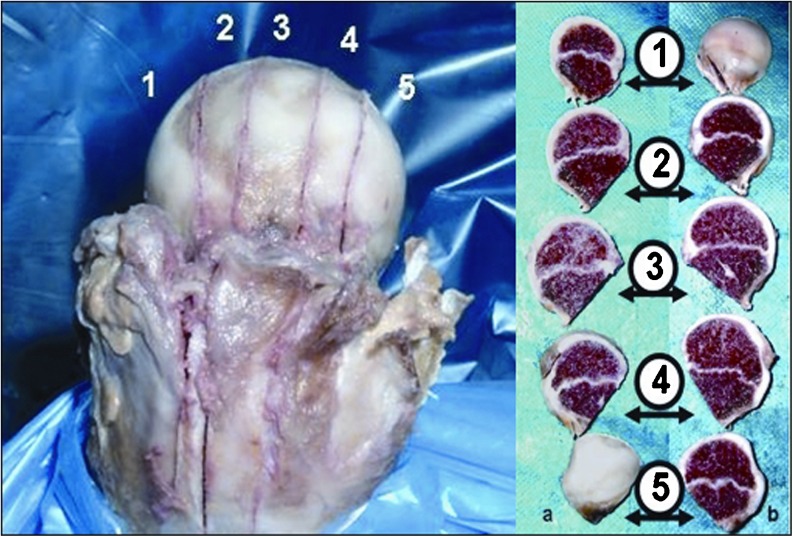
Section of a growth plate of the proximal femoral epiphysis of a 6-year-old cadaver

**Fig. 8:**
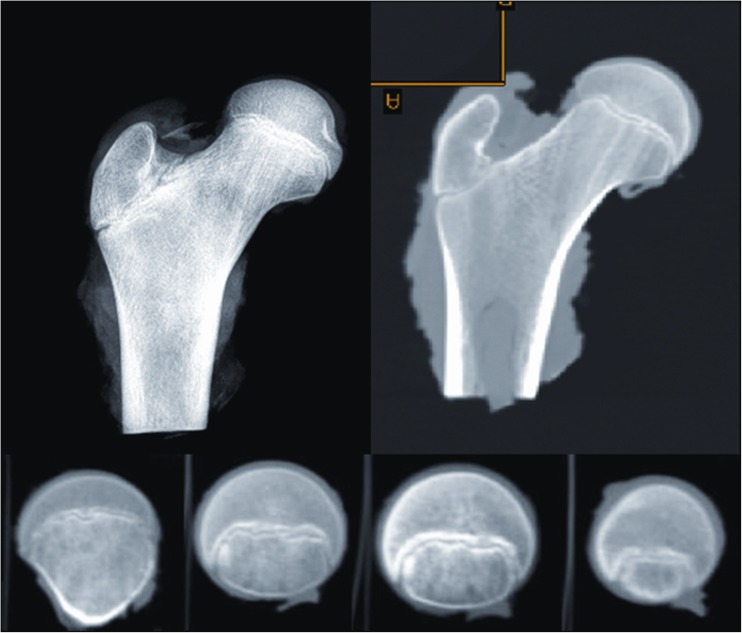
X-ray and CT image of a growth plate of the proximal femoral epiphysis of a 13-year-old cadaver

**Fig. 9:**
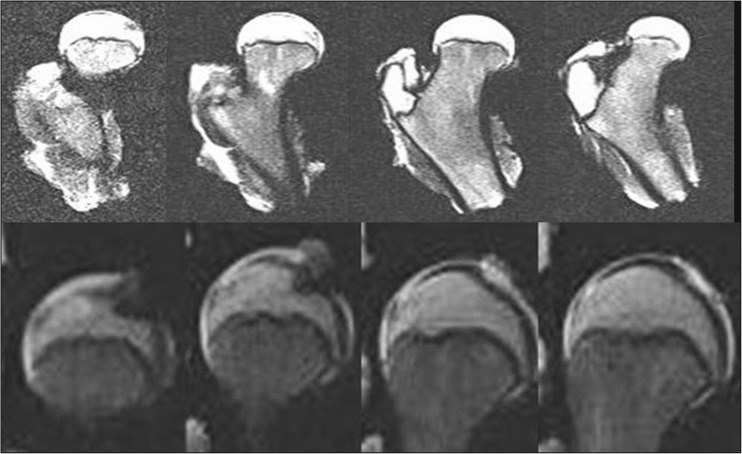
MRI scans of a growth plate of the proximal femoral epiphysis of a 13-year-old cadaver

**Fig. 10:**
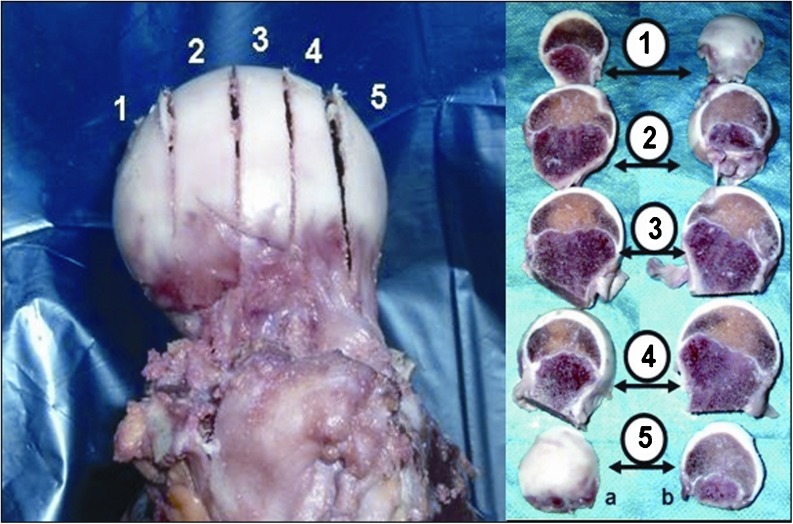
Section of a growth plate of the proximal femoral epiphysis of a 13-year-old cadaver

MRI scans also show changes in the shape of the growth plate together with much higher details in the soft tissue. In the first group of children between three and four years old the growth plate is depicted as a thick, radiological negative line that looks like a concave meniscus. The proximal epiphysis of femoral bone is ideally matched to its surface. In the second group of children the growth plate on MRI scans progressively changes its shape into a horizontal line with irregular and rugged surface. In seven year-old children the line resembles a stretched letter M. In the third group of children the growth plate looks like a horizontal line, especially within its frontal and rear fragments and the whole surface is rugged and corrugated. MRI scans of children 11–13 years old demonstrate a growth plate of a proximal femoral epiphysis as a mild arc directed upwards (Fig. 3). Minor ruggedness of its surface gradually disappears. Post-mortem examination also showed a very characteristic shape of a growth plate of a proximal femoral epiphysis. In a six year-old cadaver, examination of X-ray images demonstrates the growth plate as an irregular line with tiny ruggedness. We can see variable levels of the growth plate with two different main radiological negative lines (Fig. 4). CT scans confirm an irregular shape of a proximal femoral growth plate much better than X-ray. We can see its sinusoidal and inclusive form with abundant ruggedness that mutually interpenetrates the physis and methaphysis (Fig. 5). MRI is the best tool to demonstrate the shape of a growth plate; its irregular and sinusoidal form is shown on sagittal and planar views (Fig. 6). The proximal part of a femoral bone was cut into slices to show macroscopic details of the shape of a growth plate (Fig. 7). On X-ray images and CT scans of a 13-year-old cadaver, the growth plate is much more regular and smooth. On sagittal CT scans it has a very characteristic spherical shape (Fig. 8). On MRI scans the growth plate rises and is depicted as a consistent, smooth and arched line (Fig. 9). Macroscopic sections confirm all the findings and demonstrate the regularity, smoothness and spherical shape of a proximal femoral growth plate (Fig. 10).

## Discussion

**Fig. 11:**
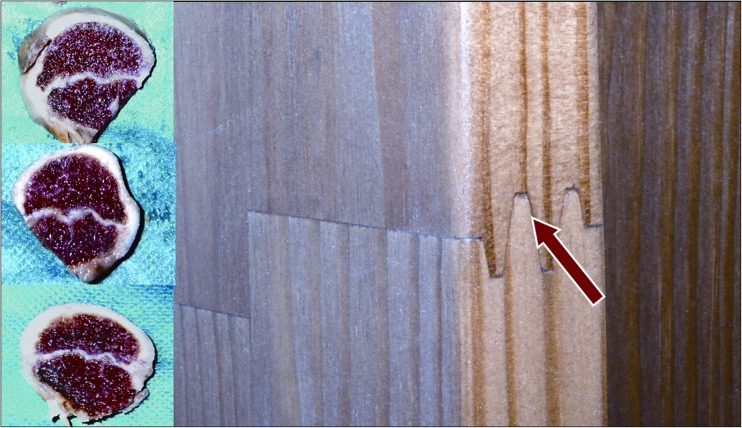
Physical resemblance between growth plate of the proximal femur and carpenter’s bonding of two pieces of wood

A growth plate is a unique structure in which stem-like cells in the resting zone differentiate into rapidly dividing chondrocytes in the proliferative zone and then finally differentiate into non-dividing chondrocytes of the hypertrophic zone. It maintains a formidable cellular organisation responsible for long bone elongation [14–16, 18]. A growth plate is a barrier for vessels but at the same time is supplied by blood from its proximal and distal side [3, 19]. Differences in a shape of the growth plate are visualised by X-ray images. In two to three year-old children a head of the femoral bone reclines on the growth plate that has a very characteristic concave shape. At the age of five a growth plate of the proximal femur loses its arc shape and starts to look like a horizontal line. Its proximal surface is irregular and rugged. Starting at the age of seven, the shape of a growth plate changes into an arc, which later resembles a stretched letter M. At the age of 11–13 a growth plate finally reaches a shape of a convex meniscus that is best depicted by MRI and CT scans. The area of the subcapital growth plate on MR images and its characteristic change together with its thinning was confirmed by Kruczyński and Wierusz-Kozłowska [20]. Mirkopulos at al., in their biomechanical study [21], present only the slope of the growth plate of a proximal part of the femoral bone but do not perform any analysis of its shape. Tiny ruggedness and pleated surface of the growth plate increase the area of connection between the head and the neck of the femoral bone. It also provides good stability and resembles a bonding of two parts of wooden material made by a carpenter (Fig. 11). Our observations were also confirmed by studies on cadavers. X-ray, CT and MRI scans emphasise differences in shape of the growth plate in children between six and 13 years old. The very characteristic sinusoidal-like shape was also shown in slice sections of the head of the femoral bone in a six year-old child. The same slice sections depict a spherical shape of a growth plate in a 13-year-old child. Changes of shape from rugged in younger children to more planar and regular in older children might lead to weakening of the connection at the level of the growth plate of the proximal part of the femoral bone.

## Conclusions

The results of our research emphasise the correlation between shape of the growth plate of the proximal femur and its influence on the incidence of SCFE. A change of the growth plate of the proximal part of the femoral bone from pleated to more spherical is an important risk factor. Together with hormonal, biochemical and genetic reasons it leads to SCFE in children ten years old and older.
